# The long and winding road of reprogramming-induced rejuvenation

**DOI:** 10.1038/s41467-024-46020-5

**Published:** 2024-03-02

**Authors:** Ali Doğa Yücel, Vadim N. Gladyshev

**Affiliations:** 1 Department of Molecular Biology and Genetics, Koc University, Istanbul, 34450 Turkey; 2 Brigham and Women’s Hospital, Harvard Medical School, Boston, MA 02115 USA

**Keywords:** Ageing, Biomarkers, Senescence

## Abstract

Organismal aging is inherently connected to the aging of its constituent cells and systems. Reducing the biological age of the organism may be assisted by reducing the age of its cells - an approach exemplified by partial cell reprogramming through the expression of Yamanaka factors or exposure to chemical cocktails. It is crucial to protect cell type identity during partial reprogramming, as cells need to retain or rapidly regain their functions following the treatment. Another critical issue is the ability to quantify biological age as reprogrammed older cells acquire younger states. We discuss recent advances in reprogramming-induced rejuvenation and offer a critical review of this procedure and its relationship to the fundamental nature of aging. We further comparatively analyze partial reprogramming, full reprogramming and transdifferentiation approaches, assess safety concerns and emphasize the importance of distinguishing rejuvenation from dedifferentiation. Finally, we highlight translational opportunities that the reprogramming-induced rejuvenation approach offers.

## Abstract (web summary)

Rejuvenation and partial reprogramming are two frontier areas in the field of aging. Here, the authors summarize advances in these fields and suggest future directions for research and therapy.

## Introduction

Over the last century, remarkable strides in medicine and public health have contributed significantly to a substantial increase in average human lifespan. Diseases associated with aging are now the leading causes of mortality worldwide in humans^1^. However, disease-focused treatments have limitations, as incidence increases in parallel for many chronic diseases, and treating one disease often makes little difference on total disease burden. As such, aging-related illnesses are more and more challenging to manage with patients’ advancing age. We now face the situation when geriatric medicine becomes progressively more impractical.

Preventive treatments targeting aging present a considerable potential as an alternative approach to combating aging-related diseases. However, to control the aging process—by either slowing it down or reversing it—one must understand the fundamental mechanisms of aging. For example, it is now well appreciated that epigenetic information is progressively lost over the lifetime of an organism^2^, disrupting cellular homeostasis. Epigenetic biomarkers of aging (aging clocks) can predict biological age through a variety of training approaches, even when based only on the variance of DNA methylation during aging^3^. Interestingly, reacquisition of the lost epigenetic information may be observed during the natural rejuvenation process that occurs during early embryogenesis as well as during cell reprogramming^4–6^. These strategies are in line with the notion of reprogramming-induced rejuvenation (RIR)^7^, a recent discovery wherein old cells can revert to a younger state upon transcription factor or chemical treatments^8,9^. RIR is commonly accomplished through partial cell reprogramming, a method in which cells transiently undergo an induced pluripotent stem cell (iPSC) reprogramming^10–16^. In this perspective, we discuss recent advances in this area, offer insights how they are related to the nature of aging and rejuvenation, and highlight potential advantages and drawbacks of this RIR and its translational potential.

It was shown that partial cell reprogramming can enhance the physiological function of human muscle stem cells^10^, ameliorate the aging mouse transcriptome and metabolome in vivo^11^, rejuvenate human dermal fibroblasts on a multi-omics level^12^, and reverse the epigenetic clock in vitro^10,12,13^. Furthermore, partial reprogramming can restore visual function in mice^14^, prevent age-related physiological changes^8,15^, and extend the remaining lifespan in wild-type mice^16^. The clinical potential of partial cell reprogramming is undeniable, but the technology has its pitfalls. We discuss potential future directions of partial cell reprogramming for therapeutic applications and biological mechanisms that support RIR. Lastly, we discuss the safety concerns of partial reprogramming and the significance of isolating RIR from dedifferentiation.

## Therapeutic potential of partial reprogramming

Partial reprogramming holds significant therapeutic potential due to its capacity for cellular rejuvenation. There are two primary approaches that may help realize the therapeutic applications of this procedure. Organismal rejuvenation is the most challenging but also the most direct approach, due to its potential to reverse aging in a manner that is independent of the identity of the cells to which it is applied. Methods for reversing aging carry the potential to generate therapies that are more efficient and effective than those aiming merely to slow down the aging processes. Organismal rejuvenation can be achieved in two ways. First, direct editing of the germline could equip each cell in the adult body with 4 F (OSKM, four Yamanaka factors), but it is currently prohibited to edit the genome of humans due to safety and ethical concerns. Another method involves delivering Yamanaka factors in the form of DNA or mRNA with the systems utilized in gene therapy. The effectiveness of this approach is currently limited by the low efficiency of existing delivery systems in certain tissues and their insufficient organ specificity^17^. However, with further advances in these methods, more precise and efficient partial cell reprogramming therapies may become feasible. Thus far, most partial cell reprogramming studies at the in vivo, full organismal level have been conducted in chimeric OSKM-inducible mice^8,11,15,18^, with an exception where the AAV9 delivery system was used to deliver the OSK factors^16^. Tissue or system-specific partial cell reprogramming is more likely to lead to positive outcomes as a therapeutic measure in the near future because partial reprogramming is expected to yield different outcomes across various tissues.

## In vivo rejuvenation of the whole organism or tissue with partial reprogramming

Administering doxycycline (dox) cyclically (2-day pulse, 5-day chase) to progeric LAKI mice carrying a Tet-inducible polycistronic OSKM cassette led to a median lifespan increase of 33% compared to control mice that received no treatment. Even after 35 cycles of dox administration, partial cell reprogramming caused neither weight loss nor mortality effects. In addition, partially reprogrammed mice apparently exhibited rejuvenation of certain cellular phenotypes, including the reduction of mitochondrial ROS and restoration of H3K9me levels^8^.

The same group applied the partial cell reprogramming procedure used in a previous study to wild-type mice, based on a long-term (7 and 10 months) and short-term (1 month) induction of OSKM factors via dox administration. Histological analyses revealed no teratoma formation resulting from the process. While this study does not provide lifespan extension data, it offers valuable insights into in vivo cell rejuvenation. Cyclic cell partial reprogramming was shown to return the transcriptome, lipidome, and metabolome of multiple tissues to a younger state. Moreover, this treatment increased skin regeneration capacity in mice^15^.

Another study demonstrated that partial reprogramming with dox-inducible OSK factors can extend the remaining lifespan of 124-week-old wild-type mice by 109% compared to untreated mice. Interestingly, this study adopted a gene therapy approach instead of using transgenic mice. OSK vectors and rtTA vectors were delivered with the AAV9 capsid to ensure maximum vector distribution in all tissues. OSK expression was achieved with the cyclic administration of dox (1-day pulse, 6-day chase). c-Myc was excluded from the cocktail to reduce the risk of teratoma formation. The frailty index score of untreated wild-type mice was 7.5 points, while partially reprogrammed TRE-OSK mice exhibited the frailty index score of 6, suggesting that the therapy may be beneficial for both healthspan and lifespan^16^.

Partial reprogramming has commonly been achieved by Yamanaka factor expression, but alternative partial reprogramming methods also exist. In particular, chemical reprogramming is an attractive method as it is a non-genetic approach, and it supports an easy delivery of small molecules throughout the body^19^. A two-chemical reprogramming procedure has shown to increase the *C. elegans* lifespan by 42.1%, and also to reduce DNA damage, ameliorate epigenetic age-related marks such as H3K9me3 and H3K27me3, partially prevent senescence and decrease oxidative stress^20^. Another study has shown the capacity to reprogram human somatic cells to pluripotent stem cells using chemicals^21,22^. Unlike OSKM-mediated reprogramming, chemical reprogramming has several steps and requires an intermediate plastic state. This could be useful for partial reprogramming studies as this approach is not as potent as the OSKM-mediated procedure, it requires several stages that could be targeted for rejuvenation, and the delivery of small molecules is advantageous, at least in the near-term, to the current gene delivery methods for therapeutic applications.

Partial chemical reprogramming of mouse fibroblasts using a 7c cocktail from Stage 1 small molecules has recently been shown to rejuvenate fibroblasts at a multi-omics scale^23^. Amelioration of mitochondrial oxidative phosphorylation, reduction in the level of aging associated metabolites, and both transcriptomic and epigenomic clocks are all indicative of rejuvenation upon partial chemical reprogramming. Interestingly, OSKM-mediated partial reprogramming downregulated the p53 pathway, whereas this pathway was upregulated upon 7c-mediated partial reprogramming, similar to its changes during the normal aging process. p53 knockout is known to significantly increase the OSKM-mediated reprogramming efficiency, meanwhile shortening the reprogramming time to a week^24^. The p53 pathway is one of key inhibitors of OSKM-mediated reprogramming^25^, and upregulation of this pathway during 7c-mediated partial reprogramming suggests that the two reprogramming types proceed through separate pathways during early reprogramming. Augmentation of the p53 pathway in mice causes stem cells to enter senescence at an earlier time point, which could potentially represent a safety issue for the in vivo 7c treatment^26^.

It was hypothesized that a set of rapid cell divisions is essential for epigenetic remodeling during OSKM-mediated cell reprogramming^27^, yet how the rate of division contributes to RIR is unknown. 7c-mediated partial reprogramming resulted in a decrease in cell proliferation, which suggests that increased proliferation is not strictly essential for cellular rejuvenation, as the opposite effect is observed during OSKM-mediated cell reprogramming^23^. Epigenetic clock reversal via 7c-mediated partial reprogramming suggests that even though the rate of cell proliferation decreases, epigenetic reprogramming can still occur efficiently. An alternative mechanism might exist for achieving epigenetic rejuvenation that doesn’t solely rely on passive demethylation of the epigenome. Further research is required to establish the role of cell proliferation and efficacy and safety of partial chemical reprogramming, determine molecular mechanisms and pathways involved, and ultimately test it at the whole-organism level.

The age and gender of an organism also impact the ability of its tissues to undergo iPSC reprogramming. For example, young female mice are less likely to undergo reprogramming than young male mice, and older mice are more susceptible to reprogramming than younger ones^28,29^. Therefore, these factors must be taken into account when considering in vivo partial reprogramming therapies. Lifespan studies can require extensive resources and time. Hence, precise biomarkers of aging and rejuvenation at organismal and cellular levels should be established. These biomarkers may include advanced multi-omic aging clocks, gene signatures, and integrated functional measures. Additionally, organismal biomarkers such as frailty index and functional tests need refinement. At the cellular level, in addition to omics-based aging biomarkers, it would be beneficial to develop aging biosensors and surface markers for profiling and imaging live cells. This integrated approach to biomarkers will not only deepen our understanding of the aging process but also potentially replace the need for extensive lifespan studies, thereby accelerating advances in the field.

## Tissue-specific partial reprogramming and its applications

Apart from whole organism rejuvenation, partial cell reprogramming can be applied to specific tissues to achieve targeted rejuvenation. A promising application of partial cell reprogramming is restoration of function, e.g. visual function. When inducible OSK-containing AAV9 was delivered to the retinal ganglion cells of old mice and mice with glaucoma via intravenous delivery, continuous expression of OSK factors led to a partially restored vision. Unlike full-organism rejuvenation experiments, continuous expression of these factors did not cause teratomas even after 10-18 months^14^. This is particularly promising for potential applications in the nervous system due to the neurons’ incomplete differentiation. Another study showed that six days of ectopic OSKM expression in the hearts of adult mice with myocardial infarction led to heart regeneration, and the infarct scar size decreased compared to that in control mice^30^. Unlike the previous study, twelve days of ectopic OSKM expression in the heart proved to be lethal for mice, suggesting that even post-mitotic tissues respond differently to partial cell reprogramming. One reason for this effect could be the c-Myc expression in the heart tissue. Although further research is needed in this field, it appears that post-mitotic tissue specific RIR may hold promise for therapeutic applications.

## Scope of partial reprogramming-mediated rejuvenation

**Fig. 1:**
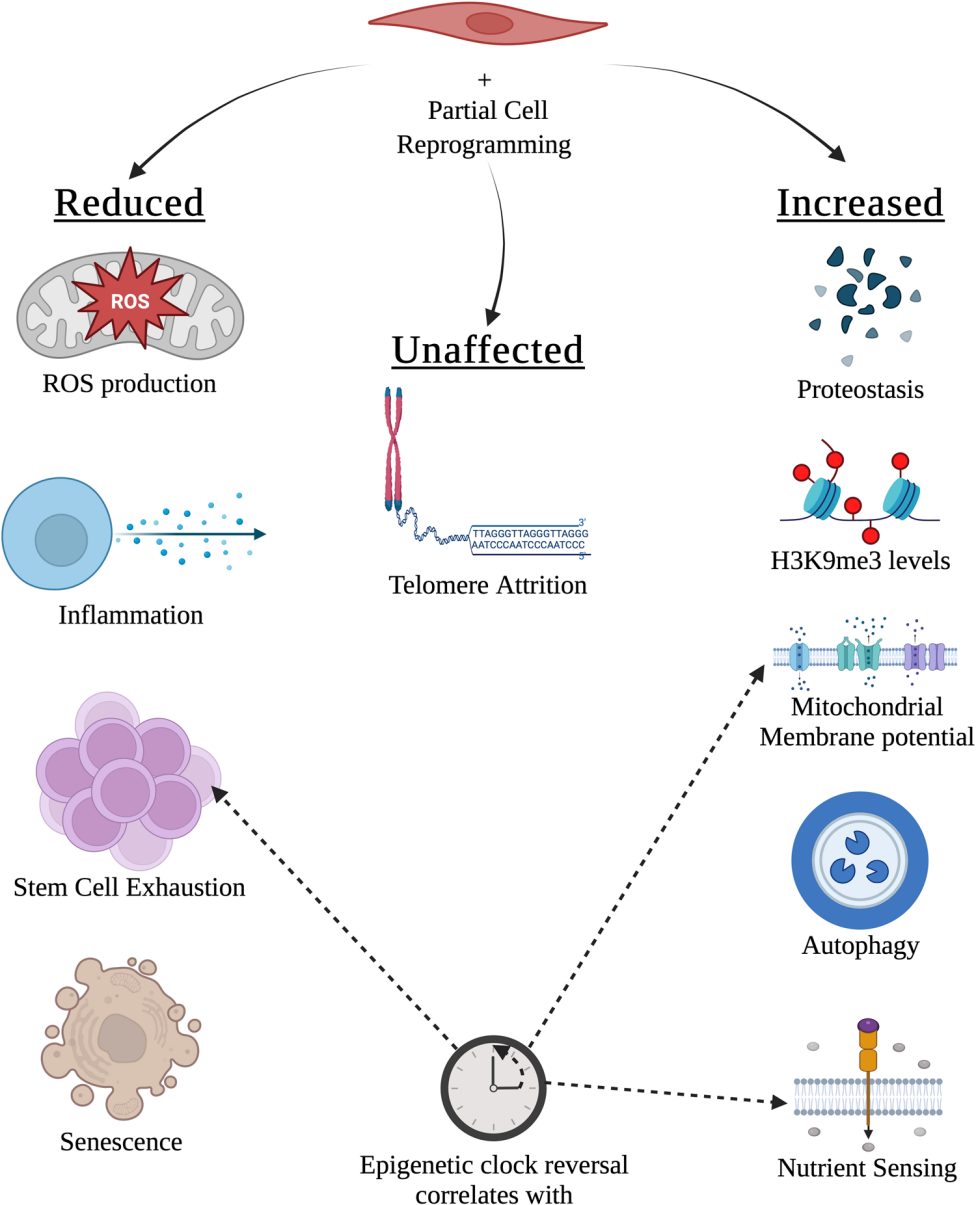
Partial cell reprogramming ameliorates the hallmarks of aging. ROS production, inflammation, stem cell exhaustion and senescence levels show a decrease, and proteostasis, H3K9me3 levels, mitochondrial membrane potential, autophagy and nutrient sensing levels show an increase in partially reprogrammed somatic cells. Increased mitochondrial membrane potential and nutrient sensing, and stem cell exhaustion strongly correlate with age reversal as measured by an epigenetic (Skin&Blood) clock. Telomere attrition is unaffected by partial cell reprogramming. Figure was created with BioRender.com.

Aging is often discussed in the context of twelve hallmarks^31^, and aging biomarkers, such as epigenetic clocks, are shown to capture some, but not all, of these hallmarks of aging. Epigenetic clock (Skin&Blood clock^32^) is associated with nutrient sensing, stem cell composition, and mitochondrial activity (Fig. 1)^33^. Partial cell reprogramming may partially reverse the biological age as reported by epigenetic clocks. This is consistent with the findings that partial cell reprogramming can restore aged muscle cell potency and revert mitochondrial aging marks by reducing ROS levels^10^. Additionally, partial reprogramming has been shown to reduce inflammation, increase autophagosome formation, increase H3K9me3 levels, and improve proteostasis, all of which are additional hallmarks of aging. Interestingly, senescence levels were lower in old endothelial cells, but not in fibroblasts^9^. On the other hand, telomere attrition was not resolved with partial reprogramming (Fig. 1), as telomerase is only activated during late cell reprogramming^34^. The impact of partial cell reprogramming on other cellular hallmarks of aging, such as altered intercellular communication and genomic instability, is currently unknown in the case of in vitro models. Although it is well-characterized that iPSC lines tend to accumulate high numbers of small-scale mutations^35,36^, the process of reprogramming is not inherently mutagenic^37,38^. However, if parental cells possess advantageous mutations prior to reprogramming, these mutations confer a competitive advantage during the reprogramming process, thereby favoring survival and dominance of such cells. This results in dominant colonies enriched with single nucleotide variants and small insertions and deletions. A significant portion of these mutations are observed in the regions associated with cell death, cell cycle, and pluripotency^37^. These observations suggest that while partial reprogramming does not increase genomic instability at the single-cell level, it might contribute to an overall increase in genomic instability within a population.

While the reversal of biological age as measured by epigenetic clocks suggests rejuvenation, these two terms should not be used interchangeably^39^. Rejuvenation can be defined as the reversal of cellular or organismal state to a state that would be found in a younger version of the organism, even though the trajectories of aging and rejuvenation may not necessarily be the same. Epigenetic, transcriptomic, and chromatin accessibility clocks may be capable of capturing certain aspects of these overall states. However, the most striking difference between epigenetic clock reversal and rejuvenation lies in their relation to causality. The first developed clocks show high correlation with age^6,40^, but their causal relationship with rejuvenation is yet to be determined, which is crucial for ascertaining their value as aging biomarkers for this type of treatment. In recent years, clocks claiming to measure biological age based on phenotypic aging and future mortality, as opposed to chronological age, have emerged^41,42^. Yet, their full applicability to rejuvenation has not been firmly established. One reason is that many clocks capture all age-related changes, whereas only some of them represent the accumulation of deleterious changes characterizing the aging process.

In this regard, the identification of CpG sites causal to aging through a Mendelian randomization approach coupled with age-related changes in DNA methylation, and the subsequent use of these sites for the development of epigenetic clocks is a new promising approach^43^. This strategy permits the construction of epigenetic clocks that may better predict longevity or a shortened lifespan. Interestingly, it was shown that commonly used epigenetic clocks are not enriched for CpG sites causally related to aging. Importantly, CpG sites that have a causal relationship with aging could be used to examine potential therapies. For example, DamAge, a clock specifically trained to capture age-related damaging changes in the DNA methylome, showed reversal of biological age, whereas AdaptAge, a clock trained to capture age-related adaptive changes, does not show this effect upon full iPSC reprogramming^43^. Further studies in this area may result in the next generation causality-informed clocks that are tuned for testing longevity interventions.

Clearly, more research is needed to understand the limits of applicability of existing biomarkers of aging for testing rejuvenation elicited by full and partial cell reprogramming. It must also be noted that even though both full reprogramming and partial cell reprogramming ameliorate cellular aging marks, there could be other factors that contribute to the observed beneficial effects in vivo. For instance, it has been shown that within a specific cell type or tissue, cells can exist in heterogeneous states with regard to biological age^44^. The overall epigenetic age of a tissue is determined by the combined contributions from various cell types, wherein certain stem cells may exhibit a younger epigenetic age than non-stem cells^33^. Tissue composition and individual aging states of cells might shift their response to partial cell reprogramming. Additionally, partially reprogrammed, biologically younger cells in an organism could proliferate more compared to biologically older cells, resulting in an overpopulation of younger cells through cell selection. Likewise, some damaged cells may be eliminated due to cell death. It has been suggested that during full reprogramming, cells harboring DNA damage are selectively eliminated through p53-mediated apoptosis^45^. This elimination of older and damaged cells could serve as a crucial selection mechanism, influencing the composition of cell population post-reprogramming. As previously discussed, parental cells with mutations conferring a survival or reprogramming advantage in genes associated with cell death, cell cycle, and pluripotency become dominant during iPSC reprogramming^37^. Although it is known which genotypic traits are favorable for iPSC reprogramming, it remains unclear if partial reprogramming selects for the same genotypic traits. Investigating this could provide insights into cell populations and their clonal selective advantage following partial reprogramming. RIR does not address the issues caused by DNA-damaged cells. On the contrary, it might amplify the tumorigenic behavior of individual cells due to clonal selection advantage. Thus, the population-level changes caused by partial reprogramming need to be thoroughly investigated to assess the benefits and risks on a larger scale.

## Partial reprogramming and rejuvenation

As mentioned above, partial cell reprogramming may rejuvenate cells and improve their physiological conditions both in vivo and in vitro. However, the underlying mechanisms of this process are not yet understood. OSKM factors activate the pluripotency gene regulatory networks (GRNs) and change the cells’ chromatin landscape during reprogramming^46,47^. It’s currently unknown whether pluripotency GRN is coupled with the rejuvenation process. During the transdifferentiation of fibroblasts to neurons and oligodendrocytes, cells preserve their aging features as well as their transcriptomic age^48^. When blood cells are directly reprogrammed into neural stem cells using non-integrating Sox2 and c-Myc, their DNA methylation age decreases compared to the donor peripheral blood cells^49^. Interestingly, their epigenetic age does not decrease close to ground zero (lowest biological age of the organism) achieving full rejuvenation, unlike the epigenetic age of pluripotent stem cells after full iPSC reprogramming. Oct4 and Sall4 expression levels do not increase at any point during this direct conversion, and the pluripotency GRN is at least partially inactive^49^. This suggests that the pluripotency GRN might be necessary for the reduction of epigenetic age of cells to the ground level, but it’s not required to partially rejuvenate the cells. Additionally, Oct4 is a master regulator of pluripotency^50^, but it is not essential for rejuvenation. Interestingly, neural stem cells after direct conversion preserve between 5.5% and 39.4% of the donor blood cells’ chronological age^49^.

These results may be interpreted in several ways:

1. The pluripotency network is not active throughout the conversion process, whereas this network is essential for epigenetic rejuvenation of cells (Fig. 2a).

**Fig. 2:**
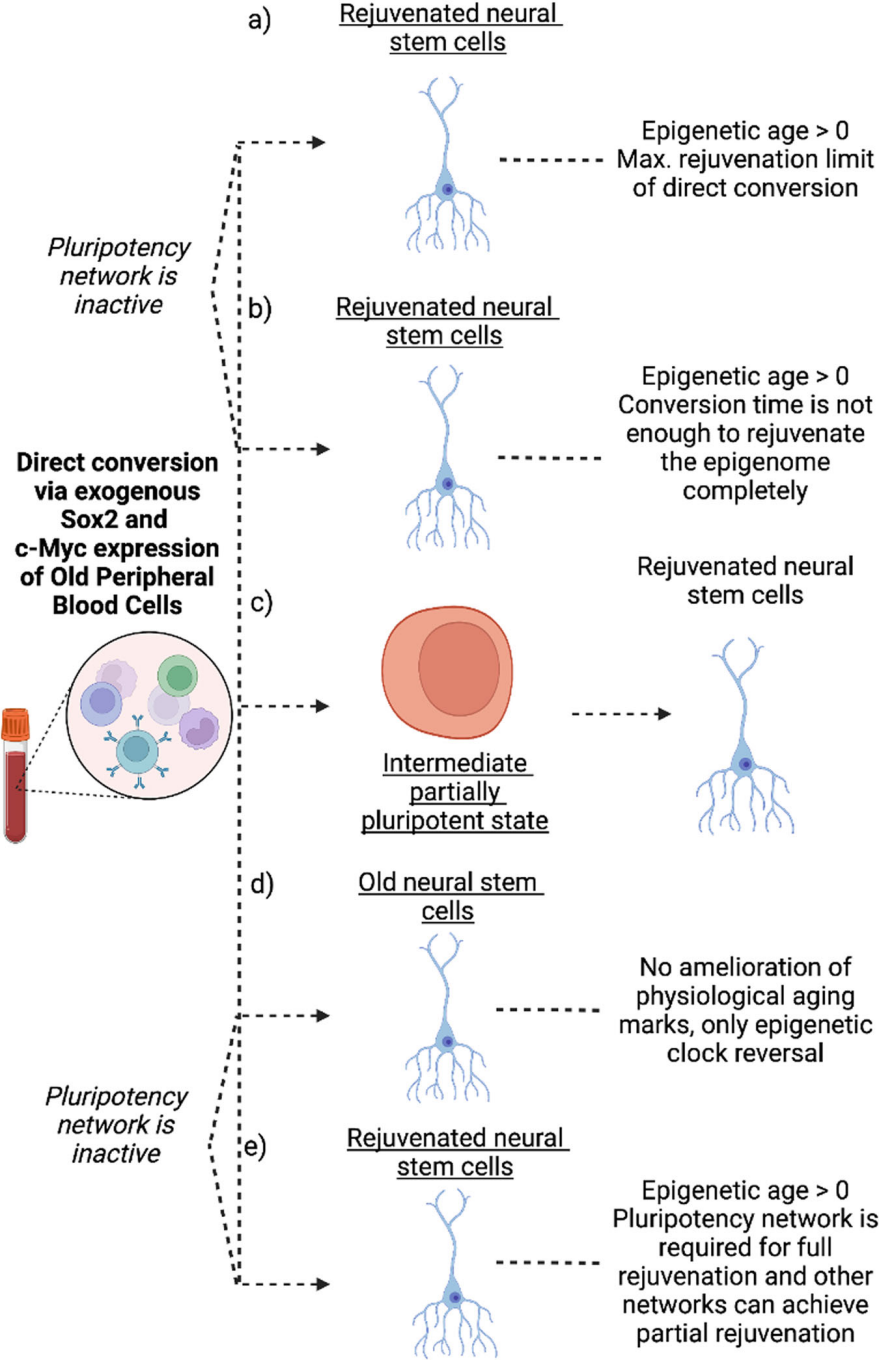
Direct conversion of old peripheral blood mononuclear cells to neural stem cells results in epigenetic clock reversal. Several explanations of the observed rejuvenation effect are possible as discussed in the text. **a** Direct conversion cannot achieve full rejuvenation. **b** Direct conversion can achieve full rejuvenation in an extended conversion period. **c** An intermediate state is required for rejuvenation where the pluripotent network is active. **d** Even though epigenetic clock reversal is observed, no age-related functional changes in the cells is observed. **e** Pluripotency network is required for full rejuvenation whereas other networks responsible during direct conversion can only partially rejuvenate. Figure was created with BioRender.com.
2. The pluripotency network is not reactivated, and rejuvenation without the pluripotency network requires a longer time. Therefore, the time length of the direct conversion procedure is not sufficient to epigenetically rejuvenate the cells (Fig. 2b).
3. The pluripotency network is partially active, and this partial activation is key for epigenetic rejuvenation (Fig. 2c).
4. There is no functional rejuvenation, and epigenetic clocks measure something other than rejuvenation, although this is unlikely due to the high correlation of the Horvath clock with functional assays in previous reports (Fig. 2d).
5. The pluripotency network is required for full rejuvenation, whereas other networks may be required for partial rejuvenation (Fig. 2e).

Another study further supports the notion that rejuvenation can be partially separated from the pluripotency GRN. This conclusion can be drawn from observations showing only a 35% average reduction in transcriptomic clock rejuvenation when genes associated with pluripotency are removed from the analysis of RIR^51^. The removal of epithelial-to-mesenchymal transition (EMT)-related genes causes a 37% average reduction in transcriptomic clock measures, potentially making them more significant to rejuvenation than the pluripotency GRN^51^. It is important to note that mesenchymal-to-epithelial transition (MET) during human fibroblast iPSC reprogramming is activated during late reprogramming whereas it is activated during early reprogramming in mouse fibroblasts^52^. This suggests that the impact of EMT genes in humans and mice could be partially different. In addition, EMT-related genes might not contribute to the human RIR. In the same study, when analyzed with the transcriptomic clock, iPSC colonies obtained with alternative 7 F reprogramming^53^ (Jdp2-Jhdm1b-Mkk6-Glis1-Nanog-Essrb-Sall4) were not rejuvenated when the Sall4 factor was removed from the reprogramming cocktail. 7F-Sall4 cocktail decreased the reprogramming efficiency significantly. Interestingly, Esrrb removal resulted in a decreased reprogramming efficiency, but Esrrb removal did not intervene with the transcriptomic rejuvenation, suggesting a potential decoupling of rejuvenation and reprogramming. Esrrb is known to be required for the completion of iPSC reprogramming by opening hypermethylated DNA regions during late Yamanaka-factor-mediated iPSC reprogramming^54^.

Recent research has also shown that Esrrb is essential for the establishment of naive pluripotency by making the epigenome available to Oct4 and Sox2^55^. The exact role of Esrrb during 7 F reprogramming is unknown since 7F-mediated iPSC reprogramming follows a different reprogramming path than Yamanaka-factor-mediated reprogramming. Given its importance in the establishment of naive pluripotency, the link between cellular rejuvenation and the naive state should be further investigated. Although rejuvenation is primarily achieved through reprogramming cells to a pluripotent or multipotent state, direct conversion could be a potential rejuvenation mechanism. By analyzing chromatin modifications, transcription factors, and DNA modifications during multiple types of reprogramming, rejuvenation pathways can be more specifically targeted. Partial reprogramming, iPSC reprogramming, and direct reprogramming provide diverse approaches for studying rejuvenation. A comparative analysis of these reprogramming techniques can offer further insights into the mechanisms and pathways of rejuvenation.

## iPSC reprogramming and partial cell reprogramming

Partial reprogramming aims to maintain cellular identity while achieving cellular rejuvenation, whereas full iPSC reprogramming aims to facilitate cellular dedifferentiation. This is the primary distinction between partial cell reprogramming and full iPSC reprogramming. Partial cell reprogramming is a relatively new field with few studies reporting on it. In contrast, iPSC reprogramming, discovered in 2006^34^, is one of the most studied concepts in stem cell biology, with a wealth of supporting literature. Given similarities in their biological mechanisms, the field of partial cell reprogramming should not disregard the existing literature on full iPSC reprogramming. This is particularly critical as it remains unknown which Yamanaka factors are essential drivers of cellular rejuvenation. Although the c-Myc proto-oncogene is one of the original Yamanaka factors^56^, its exogenous expression is not necessary for RIR and can be omitted in in vivo partial cell reprogramming studies^14,16^. Exogenous expression of Klf4, despite its role in activating key pluripotency factors, is also not necessary for full iPSC reprogramming^57^. While Sox2 is endogenously active in adult neural progenitor cells^58^, neural progenitor cells do epigenetically continue to age^33^, suggesting that Sox2 is not directly linked to rejuvenation, but a downstream pathway of Sox2 might be. As mentioned earlier, epigenetic rejuvenation can be achieved without the transcriptional activation of Oct4^49^. These results suggest that rejuvenation is not attained from overexpression of a single key factor but rather from a synergistic effect of these factors with other transcriptional networks. This also applies to full iPSC reprogramming, as Sox2-Oct4 and Sox2-Oct4-Klf4 multimeric complexes are master regulators of pluripotency and play important roles for full iPSC reprogramming^59^. Sox2, Oct4 and Klf4 have overlapping target genes and cooperate to form a functional enhancosome to create a pluripotent specific transcriptome^60^. In contrast, c-Myc is known to regulate a distinct set of gene targets and it binds to 22.4% of all promoters in the genome, regulating a wide variety of genes such as cell cycle and metabolism genes^61^. Exclusion of c-Myc from the reprogramming cocktail does not harm the rejuvenation process and suggests that target genes of c-Myc are not directly involved with the pathways of rejuvenation^14,16^.

The notion that these four factors are not directly causing rejuvenation of cells and none of them are essential for it suggests the possibility of other transcription factors primarily involved in epigenetic rejuvenation. These four factors are involved in reprogramming of the entire epigenome during naive iPSC reprogramming, which makes it challenging to pinpoint activation of which downstream pathway is primarily responsible for rejuvenation. Transcriptomic clock rejuvenation achieved by 7 F reprogramming and cellular rejuvenation achieved by multipotent reprogramming^62^ further shows that exogenous overexpression of Yamanaka factors is separable from the rejuvenation process. Therefore, the question is whether dedifferentiation is key to rejuvenation or not. Even though the current literature shows that rejuvenation is observed together with dedifferentiation, there is no established causal relation between the two concepts. If the pathways responsible for rejuvenation are also responsible for the suppression of cellular identity genes, high-throughput mutation screening for the transcription factors responsible for rejuvenation can be performed to separate cell identity suppression from epigenetic rejuvenation.

## Safety concerns of partial cell reprogramming

Although partial reprogramming is a promising and exciting technique, there are issues that hinder its therapeutic applications. The primary concern stems from the technique’s intense potency. Yamanaka factors used for partial reprogramming activate pluripotency and differentiation genes, increase proliferation, and suppress somatic cell identity^62,63^. These changes can lead to teratoma formation when these factors are continuously expressed in vivo^29,64,65^. Strictly speaking, even one fully reprogrammed cell is already too many cells at risk of teratoma, so this is a serious challenge for the intended translational potential. During epigenetic reprogramming caused by OSKM expression, suppressive epigenetic marks can be removed, which may lead to activation of oncogenes and an increase in the cancer rate. Furthermore, the coding point mutation rate is known to be elevated during iPSC reprogramming^66^ caused by clonal selection^37^, which may lead to genomic instability, also increasing the risk of cancer and heterogeneity in tissues. Moreover, continuous expression of Yamanaka factors may result in liver and intestinal failure in mice^67^. When intestinal and hepatic continuous expression of OSKM is absent during therapy, there’s a significant decrease in mortality, with 60% of mice surviving a month of continuous OSKM expression. Whole-body OSKM expression increases proliferation in tissues that are already proliferative, leading to a loss of function^67,68^. Although the contribution of hepatic and intestinal OSKM expression to these detrimental effects is not complete, it is substantial. This underscores the critical need for targeted tissue or organ-specific partial reprogramming therapies. The currently optimized method of partial reprogramming is the maturation phase partial reprogramming, which necessitates 13 days of continuous expression of Yamanaka factors in vitro. The maturation phase is proposed as the last exit prior to the point of no return for iPSC reprogramming, and significant epigenetic rejuvenation is observed on this day^12^. However, this optimized in vitro method may be highly damaging in in vivo models, as a continuous expression of Yamanaka factors for more than 2 days may have lethal effects in mice^8,67^. In vivo studies now focus on cyclic partial reprogramming, whereas in vitro studies on continuous single-cycle reprogramming, which hampers the establishment of a clear connection between these two model systems. To safely transform this method into a therapy, the impact of partial reprogramming on each tissue and system must be carefully investigated. Ethical concerns surrounding lifespan and healthspan extension therapies, particularly in the context of partial cell reprogramming, also warrant careful consideration and necessitate the creation of appropriate regulation and legislation.

## Future directions and open questions

During the aging process, certain loci increasingly come under regulation to decrease methylation variance, whereas other loci show an increase in variance due to the lack of regulation^3^. This suggests that various genes contribute to aging differently. Partial reprogramming does not provide selective rejuvenation; rather, it reprograms a wide portion of the epigenome, including parts that regulate genes not critical to aging. This lack of specificity may become evident during whole-organism partial cell reprogramming because each tissue takes a different path. Selective and targeted rejuvenation in various tissues would provide comprehensive, safe, and synergistic rejuvenation throughout the whole body. To achieve this, RIR mechanisms must be better understood in order to rejuvenate certain loci in various cell types.

Present evidence suggests that pluripotency is not inherently linked to the rejuvenation process. However, it remains unclear whether pluripotency or certain transitionary cell states can be completely uncoupled from rejuvenation. A key question to be investigated is whether certain components contributing to biological age reversal can rejuvenate the entire epigenome or only certain loci. EMT and pluripotency associated genes seem to play significant roles during rejuvenation, contributing to 37% and 35% of the transcriptomic clock rejuvenation, respectively. Both gene clusters lead to a loss in cellular identity^51^. The same analysis suggests that integrin cell interactions, collagen formation, and ECM organization gene clusters contribute to transcriptomic age rejuvenation^51^. If certain transcription factors regulating these clusters contribute to rejuvenation, they could potentially rejuvenate cells without any signs of lost cellular identity. However, it should be noted that the expression of these potential transcription factors for a prolonged period does not guarantee the rejuvenation of the entire epigenome. A similar problem arises in the case of the direct conversion of peripheral blood cells to neural stem cells^49^. If there is a limit to rejuvenation when the pluripotency network is not present, the impact of the loci that remain epigenetically old must be understood.

The low efficiency of partial cell reprogramming remains a problem, with only about 25% of cells in culture being partially reprogrammed^12^. Research in the iPSC reprogramming field is now performed to understand the factors that reduce reprogramming efficiency, such as chromatin remodelers and regulatory transcription factors. Applying the same logic to partial cell reprogramming is essential to increase the overall efficiency of the technique. It is also important to note that some key factors that decrease reprogramming efficiency are associated with cellular identity preservation^47,57^. Preservation of cellular identity must be considered while increasing the fraction of rejuvenated cells.

Additionally, persistence of rejuvenation mediated by partial cell reprogramming is an aspect that necessitates further investigation. A transcriptional analysis of adipogenic cells, reprogrammed for three days and then followed for an additional ten days, suggests that broad gene programs continue to exhibit rejuvenation^62^. However, it is crucial to note that cellular identity states of these cells post-partial reprogramming are not identical to those of their parental cells. It is essential to investigate in detail whether these cells maintain their rejuvenated states after the reprogramming period, lose all rejuvenation signs upon reverting to their initial cellular state, or experience an accelerated loss of their youthful states compared to regular cells. Therefore, the longevity of rejuvenation effects mediated by partial reprogramming continues to be a relevant and unresolved question.

There are legitimate concerns about the safety of OSK(M)-mediated partial reprogramming. To translate research in the field into clinical therapies, more research on the roadmap of partial reprogramming needs to be conducted. Furthermore, to better evaluate the results of in vivo cyclic reprogramming studies, in vitro cyclic reprogramming must be performed, and the difference between cyclic and continuous partial reprogramming must be identified.

In conclusion, while partial reprogramming holds great therapeutic potential, the real focus should be on rejuvenation research, defining its nature and ways to quantify it. Understanding rejuvenation is also key to translational success, as benefits of age reversal must be considered against risks. More research into safety and tissue-specific responses of this technique are required.
